# Mixed reality temporal bone surgical dissector: mechanical design

**DOI:** 10.1186/s40463-014-0023-9

**Published:** 2014-08-08

**Authors:** Jordan Brent Hochman, Nariman Sepehri, Vivek Rampersad, Jay Kraut, Milad Khazraee, Justyn Pisa, Bertram Unger

**Affiliations:** 1 Neurotologic Surgery, Department of Otolaryngology Head and Neck Surgery, Faculty of Medicine, University of Manitoba, GB421, 820 Sherbrook Street, Winnipeg, Manitoba, Canada; 2 Clinical Learning and Simulation Facility, Department of Medical Education, Faculty of Medicine, University of Manitoba, Winnipeg, Manitoba, Canada; 3 Faculty of Engineering, University of Manitoba, Winnipeg, Manitoba, Canada; 4 Department of Medical Education, Faculty of Medicine, University of Manitoba, Winnipeg, Manitoba, Canada; 5 Surgical Hearing Implant Program, Department of Otolaryngology - Head and Neck Surgery, Health Sciences Centre, GB421, 820 Sherbrook Street, Winnipeg, Manitoba, Canada; 6 Laboratory for Surgical Modeling Simulation and Robotics, University of Manitoba, Winnipeg, Manitoba, Canada

**Keywords:** Haptic, 3D, Virtual, Reality, Temporal, Bone, Surgery, Training, Education

## Abstract

**Objective:**

The Development of a Novel Mixed Reality (MR) Simulation.

An evolving training environment emphasizes the importance of simulation. Current haptic temporal bone simulators have difficulty representing realistic contact forces and while 3D printed models convincingly represent vibrational properties of bone, they cannot reproduce soft tissue. This paper introduces a mixed reality model, where the effective elements of both simulations are combined; haptic rendering of soft tissue directly interacts with a printed bone model.

This paper addresses one aspect in a series of challenges, specifically the mechanical merger of a haptic device with an otic drill. This further necessitates gravity cancelation of the work assembly gripper mechanism. In this system, the haptic end-effector is replaced by a high-speed drill and the virtual contact forces need to be repositioned to the drill tip from the mid wand.

Previous publications detail generation of both the requisite printed and haptic simulations.

**Method:**

Custom software was developed to reposition the haptic interaction point to the drill tip. A custom fitting, to hold the otic drill, was developed and its weight was offset using the haptic device. The robustness of the system to disturbances and its stable performance during drilling were tested. The experiments were performed on a mixed reality model consisting of two drillable rapid-prototyped layers separated by a free-space. Within the free-space, a linear virtual force model is applied to simulate drill contact with soft tissue.

**Results:**

Testing illustrated the effectiveness of gravity cancellation. Additionally, the system exhibited excellent performance given random inputs and during the drill’s passage between real and virtual components of the model. No issues with registration at model boundaries were encountered.

**Conclusion:**

These tests provide a proof of concept for the initial stages in the development of a novel mixed-reality temporal bone simulator.

## Introduction

Concern for patient safety and outcomes underscore the importance of the ever-increasing pace of development in medical simulation.

**Figure 1:**
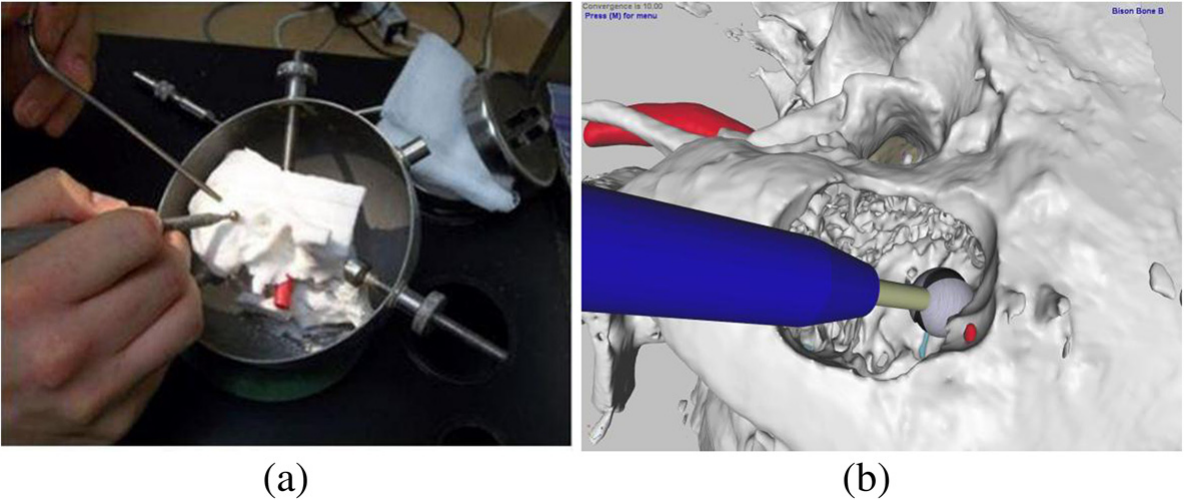
**Current state of temporal bone surgical simulation. a)** 3D printed temporal bone model and **b)** Stereoscopic Bimodal Haptic Graphic Virtual Temporal Bone Simulation. Both simulations were developed at the University of Manitoba, Laboratory for Surgical Modeling Simulation and Robotics.

Simulators are now ubiquitous in training [Figure 1]. Interactive computer-driven simulations provide a safe training environment for learning anatomy and procedures. Several haptic temporal bone simulators are currently available [1]–[5]. A haptic device is a robotic system designed to apply forces through an end-effector. As a virtual tool comes into contact with virtual tissues, reaction forces are simulated. Unfortunately, owing to fundamental limitations in mechanical design, existing haptic simulations are unable to realistically reproduce the vibration and contact forces experienced during surgery.

Printed models provide another approach to temporal bone simulation and can be created from virtual models using a variety of techniques. A plaster based printer (Z-Corporation, Rock Hill, SC) uses digitized data and places successive layers of material, building a physical model (additive manufacturing). The printed models provide a realistic sense of hardness, but are ill-equipped to present soft tissues [6].

Each of the former simulations have been generated in our Lab [7], however, there is an opportunity to address the limitations in both with an entirely new mixed-reality paradigm. Mixed reality (MR) techniques fuse digital data with the human perception of the real environment. The MR simulation will combine a physical printed bonemodel with a virtual soft-tissue model. The virtual model will employ a haptic device (HD^2^, Quansar Corporation, Markham, Ontario) that provides real-time contact force representation. This device is a 6 Degree of Freedom (DOF), 10 newton per axis manipulandum. The haptic device will generate forces encountered during interaction with virtual soft tissues, such as decompression of a sinus or dural plate, as well as permit metric assessments. The resulting MR model should provide a platform that is capable of both the realistic force-feedback and visualization surgical trainees require.

Developmental steps in generation of a MR Simulator:

1. Segment DICOM data

2. Mechanical merger of a six DOF haptic device with otic drill

3. Generate a virtual haptic model

4. Generate a 3D printed temporal bone model

5. Develop registration techniques to co-locate printed and virtual models

6. Develop control algorithms for virtual force-feedback interaction

7. Validate system

The challenges in creation of novel MR Temporal Bone Simulation are considerable. This paper addresses just one aspect, specifically the mechanical merger of a six DOF haptic device with an otic drill system (Medtronic, Minneapolis MN). This is accomplished with a custom gripper mechanism. In this structure, the drill becomes the haptic end-effector.

In the MR simulation, a user must be able to employ the drill as they would in surgery. The merged haptic drill device needs to successfully navigate three different contact conditions during device operation: movement in free space, contact with rapid-prototyped bone, and contact with virtual soft tissue. Throughout these conditions and in transitions between them, the user should feel only the weight of the drill. Further, real and virtual forces need be displaced to the drill tip from the normative mid-shaft of the haptic end-effector.

## Methods

**Figure 2:**
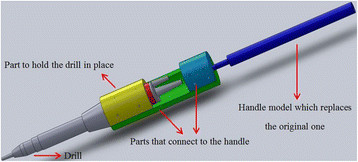
**Work assembly of haptic gripper.** Solid-Works model of the assembled gripper; permitting union of the haptic device and otic drill.

We have developed a custom fitting for the haptic device which holds an otic drill. The fitting consists of several pieces [Figure 2] and secures the drill, which then acts as the haptic end-effector.

**Figure 3:**
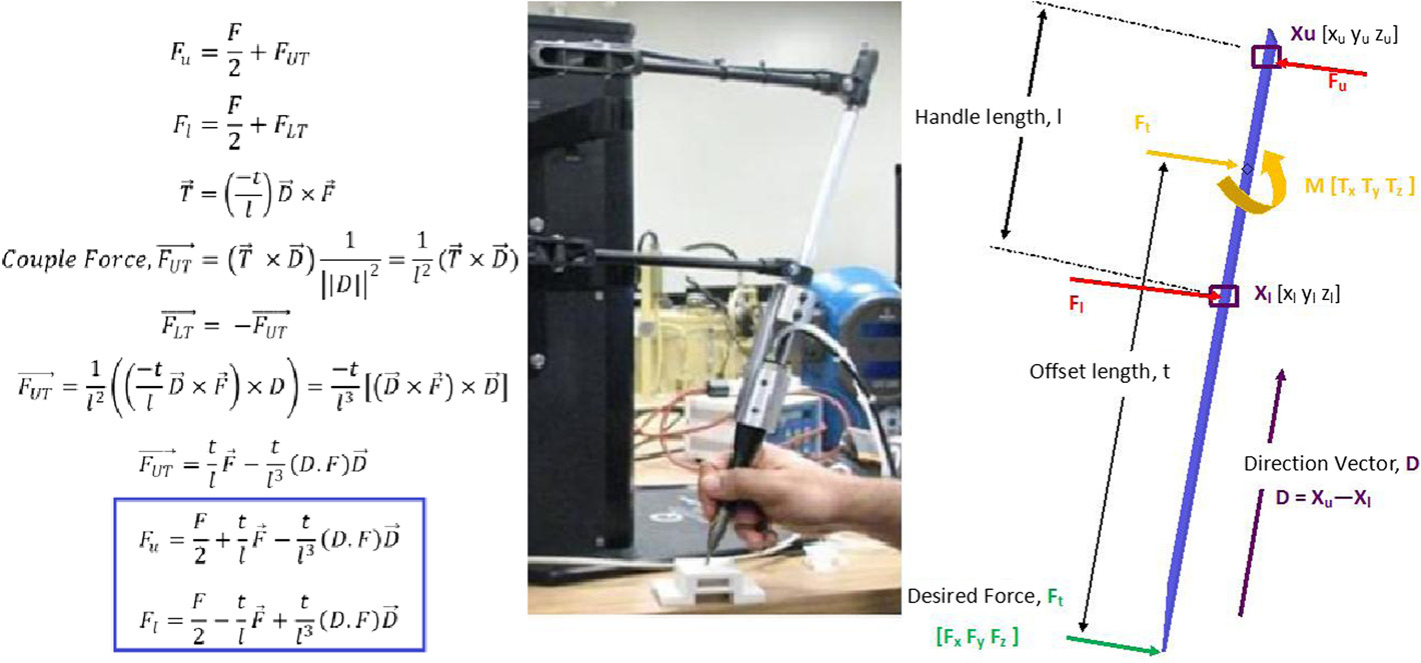
**Converting haptic interaction point to drill tip from mid-wand.** Haptic contact forces should be felt at drill tip rather than at the mid-point of the haptic wand. This necessitates the following changes to the forces created by the haptic device, where *x* is the cross product, *t* the distance between drill tip and haptic wand mid-point and *l* is handle length.

Contact forces with virtual objects are normally applied at mid-point of the haptic end-effector. A user would feel that contacts are coming from this location, at the point where the end-effector is grasped. In order for the user to feel that the forces are the result of drill interactions with the virtual environment, the contact forces must be re-located to the tip of the drill. This is accomplished in software, adjusting the haptic device force and torque outputs to take drill length, position and orientation into account [Figure 3]. The result is natural-feeling force-feedback during bone drilling.

Software was also developed to offset gravitational forces permitting the user to feel only the weight of the drill and not the custom fitting. The software guides the haptic device motors to dynamically generate an upwards force, equal to that of the drill fittings, compensating for their additional weight.

A series of tests were performed to ensure the operability and stability of the re-designed system under normative conditions. The first set of tests determined the ability of the system to generate sufficient power to offset the weight of the custom end effector.

A second set of tests determined the effectiveness of the system in position control mode during disturbances from the user.

**Figure 4:**
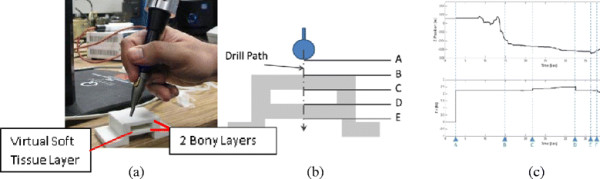
**Stability assessment while dissecting a simple mixed reality model. a)** represents a mixed reality model with HD^2^, otic drill and gripper assembly. Note the simple 3-layer rectangular printed model. **b)** shows the vertical path of the drill through the model with labels at interfaces between real and virtual modes of operation. At position A, the drill is in free space, with only gravity compensation forces generated by the haptic device. This results in a steady 2.5 N z-axis force. The user moves the drill down to interact with the printed model surface at location B. From B to C, the user encounters real forces from drilling in addition to the constant gravity compensation forces. From C to D the drill is between real bone model layers and a virtual soft tissue force is generated by the haptic device, increasing with depth of penetration. From D to E, the drill is engaged with the second real bone model layer and the virtual force is off. At E, the drill enters the free space below the second bony layer where only gravity compensation forces are present. **c)** shows a recording of the drill’s z-axis position in metres (upper axis) and force in Newtons (lower axis) generated by the haptic device. The system remains stable throughout, without high-frequency or underdamped oscillations at boundaries between models.

The third set of tests examined system stability during drilling with a simple MR model [Figure 4]. The model consists of two drillable rapid-prototyped layers, separated by a free-space. Within the free-space, a virtual spring force model is applied by the haptic system. Force feedback increases steadily with depth of penetration through the free space. The real and virtual models are registered/co-located to each other so that, as the drill penetrates the first real layer of the model, only gravity compensation is engaged. When the empty space below the first layer is encountered, the virtual spring model is additionally employed. Once the drill has passed through the empty gap, it again encounters the real model and only gravity cancelation is engaged.

## Results

**Figure 5:**
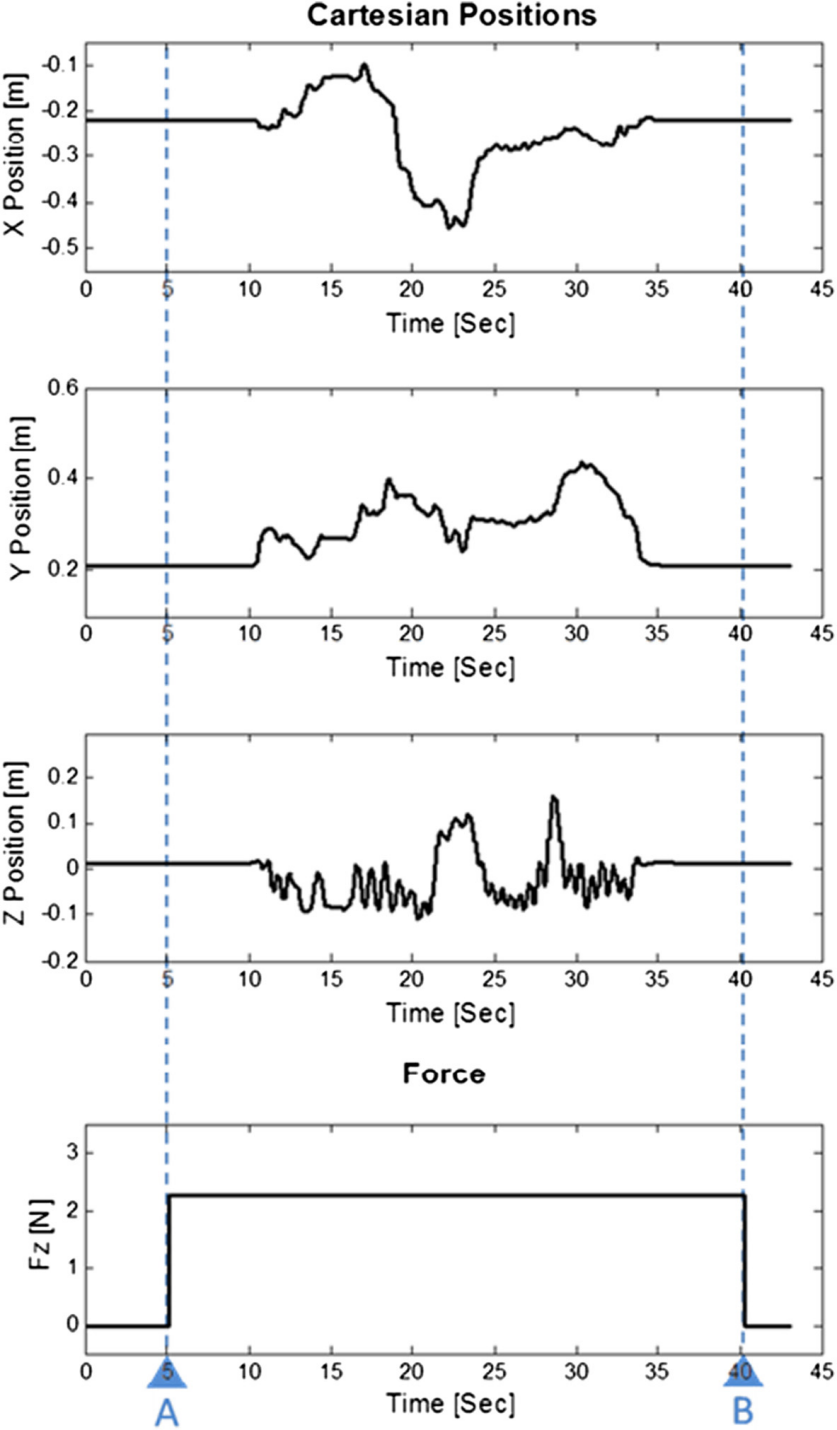
**Operation with gravity compensation throughout the device work space.** These graphs show user manipulation of the mixed-reality system within its nominal workspace (x, y, z position in meters - top three axes). Gravity compensation for the gripper is provided by z-axis forces (F_z_ in Newton- bottom axis) and is present between times A and B. No instability is seen, as manifested by the absence of high frequency or undamped oscillations in the device position recordings.

Gravity cancellation of the gripper assembly was effective without visible drift, throughout the nominal device workspace [Figure 5]. A force of 2.5 N in the z-axis, and 0.1 Nm pitch-torques and 0.02 Nm roll-torques were required; less than the maximum continuous output capabilities of the device (7.67 N z-axis and 0.948 Nm). The system remained stable during random input from experimenters during operation of the otic drill up to its maximum of 42,000 rpm.

Stability of the system during the drill’s passage through a MR model was monitored [Figure 4]. No issues with registration or instability at model boundaries were encountered.

## Discussion

The integration of printed and virtual simulation provides a platform which can represent otic drill forces as well as contact of the drill (or potentially other instruments) with embedded soft tissue structures and facilitate metric assessments. A MR system can complement existing simulations in creating a realistic and reproducible surgical training platform, which can be used to teach/assess trainees. The system permits mistakes and explorations of technique and represents an immense opportunity to improve patient safety.

Several significant challenges remain to be addressed. Highly accurate co-location of the virtual and printed simulations is requisite and will be complicated by the need to change the position of the simulation during dissection, as in real surgery. The virtual force algorithms need to be modified for the HD2 haptic as they were initially developed for a 3 DOF device. Further, investigations of construct and concurrent validity are necessary.

These tests provide evidence for the effective mechanical and software design of a novel system, integrating an otic drill with an existing haptic device. The new system provides gravity compensation and operational stability during interactions with a simple mixed-reality model.

## Conclusion

These tests provide a proof of concept for the initial stages in the development of a novel mixed-reality simulator.

## Research support

Medtronic: Donation of Otic Drill System

NSERC-Engage Grant (Industry Collaborator: Quanser)

Health Sciences Centre Foundation

University of Manitoba Faculty of Medicine
